# Design and Realization of MEMS Heart Sound Sensor with Concave, Racket-Shaped Cilium

**DOI:** 10.3390/bios12070534

**Published:** 2022-07-18

**Authors:** Yuhua Yang, Bo Wang, Jiangong Cui, Guojun Zhang, Renxin Wang, Wendong Zhang, Changde He, Yirui Li, Pengcheng Shi, Shuotong Wang

**Affiliations:** State Key Laboratory of Dynamic Measurement Technology, North University of China, Taiyuan 030051, China; wang781897@163.com (B.W.); jgcui@nuc.edu.cn (J.C.); zhangguojun1977@nuc.edu.cn (G.Z.); wangrenxin@pku.edu.cn (R.W.); wdzhang@nuc.edu.cn (W.Z.); hechangde@nuc.edu.cn (C.H.); liyirui163@163.com (Y.L.); shipengchengzb@163.com (P.S.); wangshuoton@163.com (S.W.)

**Keywords:** bionic, MEMS, sensor, stethoscope

## Abstract

The biomedical acoustic signal plays an important role in clinical non-invasive diagnosis. In view of the deficiencies in early diagnosis of cardiovascular diseases, acoustic properties of *S*_1_ and *S*_2_ heart sounds are utilized. In this paper, we propose an integrated concave cilium MEMS heart sound sensor. The concave structure enlarges the area for receiving sound waves to improve the low-frequency sensitivity, and realizes the low-frequency and high-sensitivity characteristics of an MEMS heart sound sensor by adopting a reasonable acoustic package design, reducing the loss of heart sound distortion and faint heart murmurs, and improving the auscultation effect. Finally, experimental results show that the integrated concave ciliated MEMS heart sound sensor’s sensitivity reaches −180.6 dB@500 Hz, as compared with the traditional bionic ciliated MEMS heart sound sensor; the sensitivity is 8.9 dB higher. The sensor has a signal-to-noise ratio of 27.05 dB, and has good heart sound detection ability, improving the accuracy of clinical detection methods.

## 1. Introduction

Cardiovascular disease has become a frequent and common disease that endangers human health. Therefore, the prevention and diagnosis of cardiovascular diseases represented by coronary heart disease, especially through the research of non-invasive diagnosis technology based on modern information technology, has become an important topic of clinical diagnosis research. Auscultation is an indispensable, routine examination method for clinical diagnosis. The French doctor Laennec invented the stethoscope in 1816 so that “indirect auscultation” became possible, and disciplines such as cardiac auscultation were established, which greatly promoted the development of medicine [1,2].

Biomedical signals, especially human biomedical signals, are generated by a variety of living organisms. Like other creatures in nature, each organ in the body is moving in accordance with specific laws. The vibration of movement produces corresponding sound information, whereas different sound information carries different physiological and pathological characteristics of each related organ. After continuous development and improvement of medical auscultation, the 3M Littmann company in the United States made a significant breakthrough concerning medical auscultation in 2000, and applied the developed electronic stethoscope to auscultate by using electronic technology for the first time [3,4]. With the constant improvement and development of science, the stethoscope has become electronic, intelligent, and portable. The cardiac sound sensor, based on the high-density microphone, was developed by A. Mashubabu et al. in 2016 [5]. In 2017, a piezoelectric PVDF-membrane heart acoustic sensor was fabricated by S. Afattah et al. An innovative interferometric cardiac sound sensor was invented by R. Martinek et al. in 2018 by utilizing optical fiber [6]. Exploiting the MEMS technology, Zhang Guojun et al. at North University of China developed a bionic sensing unit for detecting heart sounds in 2019, where the piezoresistors and beams were used as the core, marking the diversified advancement in cardiac sound auscultation approaches [7,8]. Taking the 3M electronic stethoscope as an example, piezoelectric materials play a role as the sensing element. When the piezoelectric material is deformed, polarization is generated inside it; meanwhile, charges with opposite signs are generated on its two surfaces. However, the charges generated are easy to leak and insensitive to low-frequency signals. In addition, the sensitivity of the piezoelectric sensor is determined by the thickness of the piezoelectric vibrator, and the piezoelectric material is also likely to be damaged in the manufacturing process [9,10]. With the development of MEMS technology, the application of MEMS technology to medical auscultation has become a trend [11].The heart sound sensor based on MEMS technology realizes the acquisition and capture of the acoustic signal through the piezoresistive effect, and a reasonable acoustic package is designed to effectively reduce the propagation sound loss of the heart sound signal from the human tissue to the sensor receiving process.

Heart sound, which is in the cardiac cycle, is a mechanical vibration that is the result of myocardial contractile function and diastolic or blood flow shock to the ventricular wall. It passes around the chest wall and the weak vibration signal is generated. The recognition and analysis of the heart sound signal have practical application values and clinical significance in the diagnosis of cardiovascular disease, and their accuracy and reliability are directly related to the diagnosis and treatment effect. When the heart or cardiovascular disease has not progressed to ECG abnormalities, pain and other symptoms, the murmurs and aberrations of the heart sounds are reliable information for early diagnosis. Heart sound diagnosis is non-invasive, fast, convenient, and economical, and can be used for extensive general surveys and preventive diagnosis. It can also be used to observe the dynamic process of the heart.

Medical bionics studies the structure, function, and working principle of the organism by imitating its life characteristics and organizational structure characteristics, and transplants these principles into medical technology. With the in-depth study of medical bionics, we find that medical bionics helps to make our medical measures closer to an ecological nature, and promotes the organic combination of traditional medicine and modern medicine and the development of the medical cause.

## 2. Principles and Methods

### 2.1. Principle and Structure

The MEMS heart sound sensor, structured with a cilium and beams, mainly mimics the structural pickup mechanism of 3D fiber bundles in otic hair cells of humans [11]. Combined with the bionic principle, piezoresistive effect, and MEMS technology, the new bionic MEMS microstructure is composed of a cilium and high-precision cantilever beams. The bottom of the cilium is fixed at the central connection of the beam members; meanwhile, varistors with identical resistance are arranged below the maximum stress zone of cantilever beams, which form a Wheatstone bridge (*V_CC_* means the input voltage of the bridge and *V_out_* suggests the output voltage).

When sound waves are applied to the biomimetic cilium, the biomimetic cilium oscillates, deforming the cantilever beams. Finally, the deformation of the cantilever causes a change in the resistance of the varistor placed on the cantilever surface. The mechanical deformation is transformed into voltage output via the Wheatstone bridge. [12]

**Figure 1:**
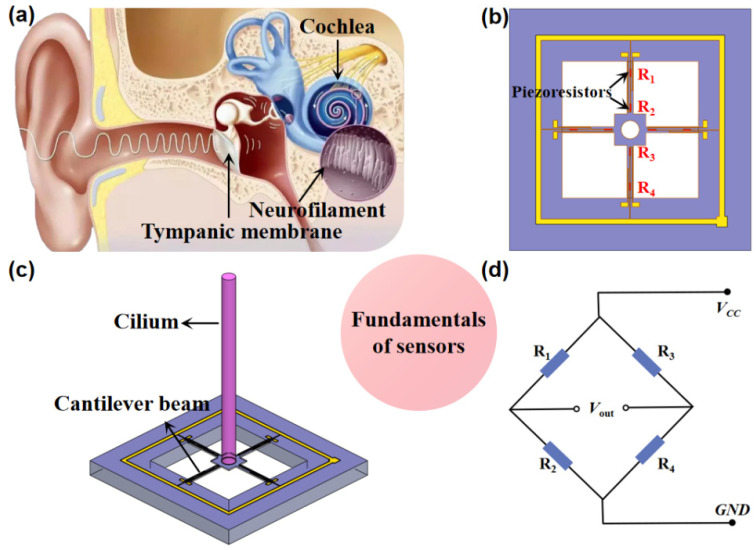
Principle of bionic microsensors. (**a**) Human ear’s sensory organ structure; (**b**) analysis diagram of microstructure; (**c**) microstructure of bionics; (**d**) Wheatstone circuit diagram.

The human ear’s sensory organ structure is shown in Figure 1a. An analysis diagram of the microstructure is shown in Figure 1b and the microstructure of bionics is shown in Figure 1c. *R*_1_, *R*_2_, *R*_3_, and *R*_4_ form a set of Wheatstone bridges, as shown in Figure 1d. At this time, the output voltage of the Wheatstone bridge is shown in (1):

(1) $$V_{out}\frac{\left(R_{1}+\Delta R_{1}\right)\left(R_{4}+\Delta R_{4}\right)-\left(R_{2}-\Delta R_{2}\right)\left(R_{3}-\Delta R_{3}\right)}{\left(R_{1}+\Delta R_{1}+R_{2}-\Delta R_{2}\right)\left(R_{4}+\Delta R_{4}+R_{3}-\Delta R_{3}\right)}V_{cc}$$

In Formula (1), the output voltage of the X circuit of the *V_out_* bridge, *V_cc_*, is the input voltage, and ∆*R* is the resistance change. Where *R*_1_ = *R*_2_ = *R*_3_ = *R*_4_ = *R*, the above formula can be simplified as shown in (2):

(2) $$V_{out}=\frac{\Delta R}{R}V_{cc}$$

The resistance change is related to the stress on the cantilever beams, which is shown in (3):

(3) $$\frac{\Delta R}{R}=\pi_{l}\sigma_{l}$$

In Formula (3), *σ_l_* is the longitudinal stress component, and *π_l_* is their longitudinal piezoresistive coefficient. In order to improve the sensitivity of the sensor, it is necessary to make the sensor output as high as possible. Placing the varistor in the maximum linear stress region of the cantilever beams can effectively improve the sensitivity of the sensor.

**Figure 2:**
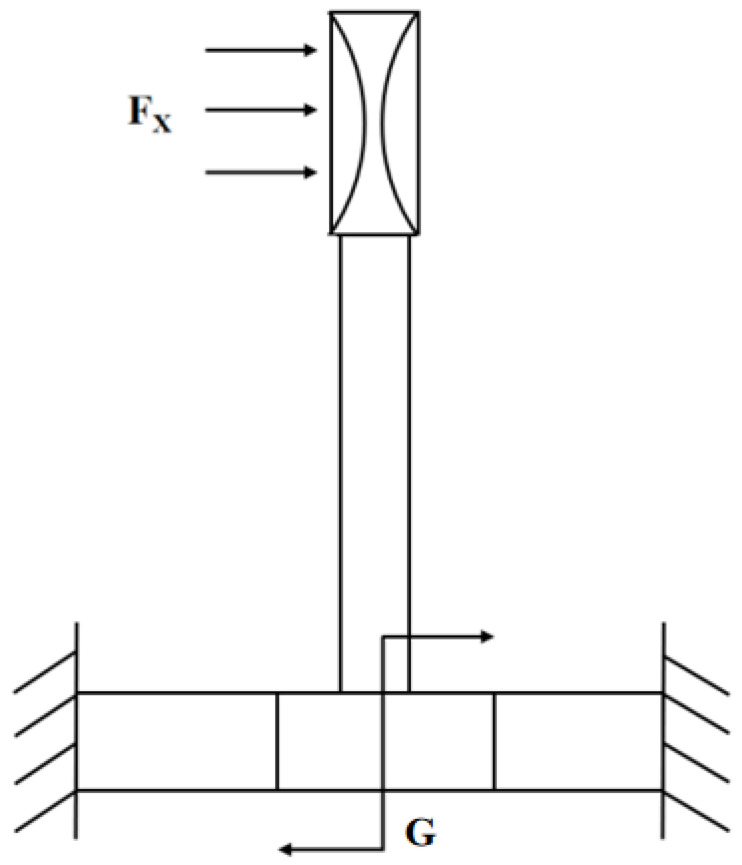
Mechanical analysis of microstructural model.

Figure 2 shows the force analysis diagram of the microstructure cross-section. When the heart sound signal acts on the bionic cilium, the bionic cilium deviates slightly, twisting to drive the cantilever beams to twist. The bionic cilium transforms the effect of sound pressure into the torques of the cantilever beams. The result of the force analysis of the cantilever beams can be obtained as shown in (4) [13,14]:

(4) $$\sigma_{\left(x\right)}=\pm \frac{L^{2}+3aL-3x\left(a+L\right)}{\frac{2}{3}bt^{2}\left(L^{2}+3aL+3a^{2}\right)}PSh\pm \frac{PS}{bt}$$

In Formula (4), *x* is the length of the cantilever beams, *P* is the pressure on the cilium receiving the heart sound signal, *S* is the area of the cilium receiving the heart sound signal, *h* is the height of the center of gravity of the cilium structure, and other parameters are shown in Table 1. It can be seen that the stress on the cantilever beams is proportional to the area that receives the heart sound signal, and inversely proportional to the width and thickness of the cantilever beams. The cilium is an important structure for the sensor to receive heart sound signals.

The working bandwidth of the sensor is mainly reflected by its characteristic frequency. The characteristic frequency of a system is mainly related to the mass m and elastic coefficient *k* of the cilium. The calculation formula of the characteristic frequency is shown in (5):

(5) $$f=\frac{1}{2\pi }\sqrt{\frac{k}{m}}$$

It can be seen from Equation (5) that the characteristic frequency of the sensor is inversely proportional to the cilium’s own quality. As the radius and length of the cilium increase, the detection bandwidth of the sensor also decreases. According to clinical research, the frequency of most physiological information of the heart sound signal is distributed at 20~600 Hz. Therefore, on the premise of satisfying the working bandwidth of the heart sound sensor, the low-frequency sensitivity of the heart sound sensor can be improved. At the same time, in order to avoid affecting the deflection of the central mass and to consider the stability of the sensor, the diameter of the cilium at the bottom should not exceed the side length of the mass. Finally, this paper proposes the integrated concave bionic ciliated MEMS heart sound sensor. The concave design of the bionic cilium improved the receiving area and focused the performance of the bionic cilium on the sound signal, improving the low-frequency sensitivity of the MEMS heart sound sensor.

### 2.2. Design and Optimization of Structure

**Figure 3:**
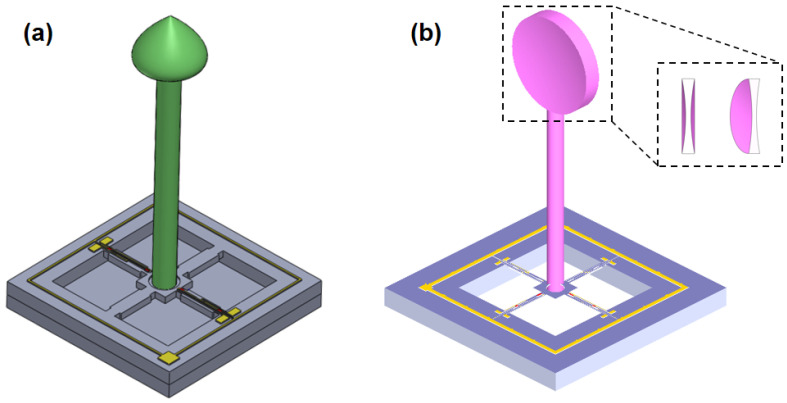
(**a**) Traditional ciliated global microstructural model; (**b**) Integrated microstructure model of concave cilium.

Under the previously described theory, in order to realize the low-frequency and high-sensitivity characteristics of the heart sound sensor, a concave ciliated heart sound sensor was designed in this study. Compared with the traditional bionic ciliated MEMS heart sound sensor, as seen in Figure 3a [15], the concave ciliated microstructure in Figure 3b improved the sensitivity of the microstructure by increasing the stress area, and effectively improved the low-frequency and high-sensitivity of the heart sound sensor.

For determining the optimal structure size of the cilium and improving the heart sound sensor performance, the structure and size of the cilium were parameterized in COMSOL.

**Figure 4:**
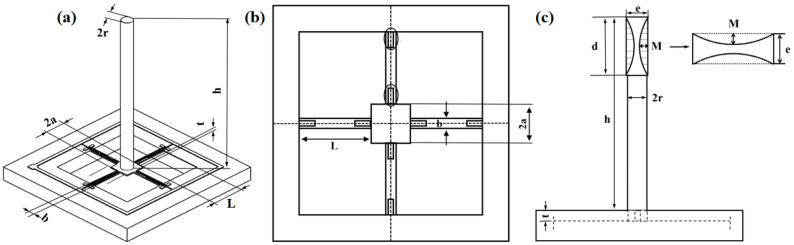
(**a**) Microstructure model for sensor; (**b**) integrated microstructure model of concave cilium; (**c**) microstructure model of concave cilium.

Figure 4c shows the integrated concave cilium structure model, where r means the cilium radius; h suggests the cilium height; d represents the concave cilium diameter; M indicates the concave depth; and e denotes the concave cilium width. Restricted by the manufacturing technique, the size optimization scopes for the cilium structure are: 0.145 mm ≤ r ≤ 0.165 mm, 3 mm ≤ h ≤ 5 mm, 1.5 mm ≤ d ≤ 2.2 mm, 0.34 mm ≤ e ≤ 0.4 mm. Material attributes are shown in Table 2.

**Figure 5:**
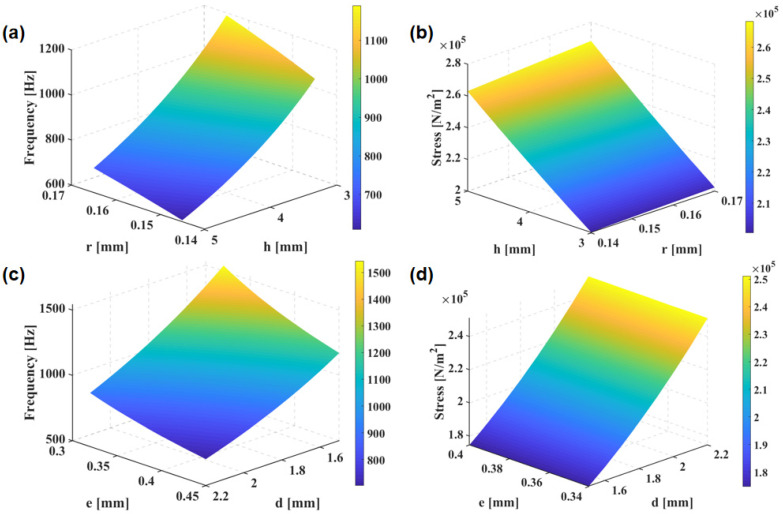
(**a**) Effects of radius r and height h on the first-order solid frequency; (**b**) effects of height r and radius h on the maximum beam stress; (**c**) effects of the concave diameter b and the concave cilium width t on the first-order solid frequency; (**d**) effects of concave diameter b and concave cilium width t on the maximum stress on the beams.

**Figure 6:**
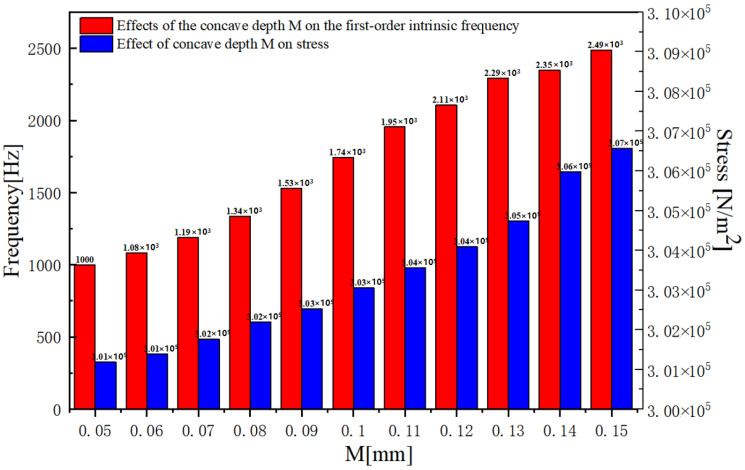
Effects of the concave depth M on the first-order intrinsic frequency and effect of concave depth M on stress.

Through the finite element simulation calculation carried out by the iterative analysis method, Figure 5a,c exhibit the increase in natural frequency as r increases, and the decrease as h, d, and e increase. As is clear from Figure 5b,d, with increasing r, h, and d, the maximum stress on the cantilever beams increases, whereas with increasing e, the stress shows a decline. Figure 6 shows that with the increase in the concave depth M, both the first-order solid frequency and stress of the microstructure increase. Considering the actual requirements of the working frequency band and sensitivity, the size of the integrated concave ciliated microstructure was finally determined: the concave depth is 0.1 mm; cilium height is 5.7 mm; cilium radius is 0.165 mm; concave radius is 1 mm; and overall concave width is 0.34 mm.

**Figure 7:**
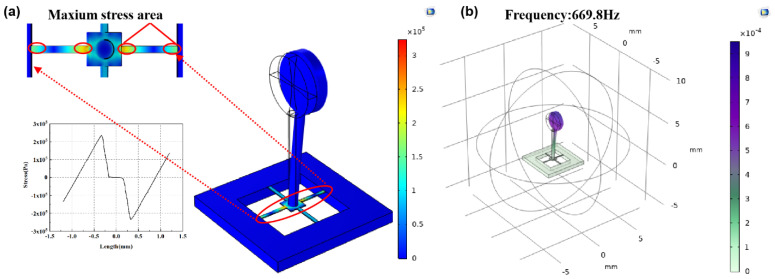
(**a**) Centralized stress simulation of the cantilever beam microstructure. (**b**) First-order natural frequency of concave microstructure.

The stress analysis of cantilever beams is shown in Figure 7a and the first-order natural frequency in concave cilium microstructure in liquid is seen in Figure 7b.

### 2.3. Sensor Microstructure Manufacturing Process

**Figure 8:**
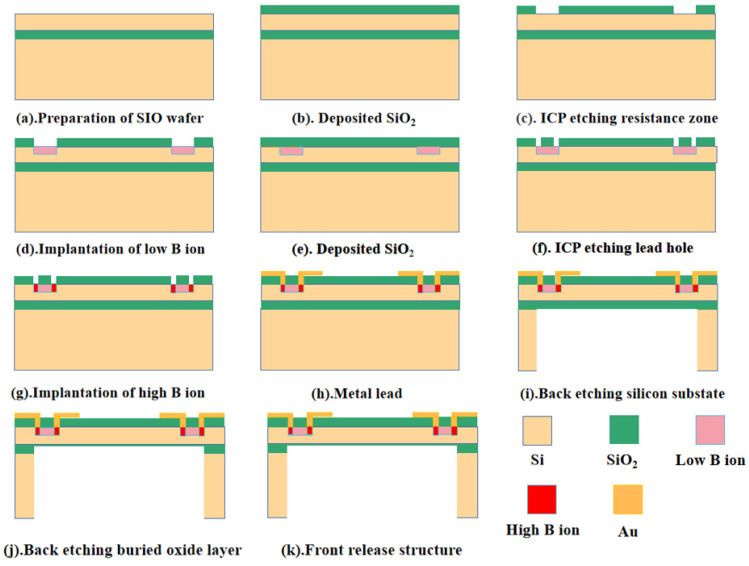
Schematic diagram of main process flow of sensor microstructure.

The MEMS processing technology was adopted to treat the microstructure of the bionic MEMS heart sound sensor designed in this paper. The design of the process flow was combined with the specific actual process conditions to formulate the process flow design method, which mainly included: lithography, oxidation, doping, bonding, etching, ion implantation, deposition, dry etching, wet etching, metal sputtering, scribing, cleaning, and other processes. The main process flow is shown in Figure 8 below.

## 3. Sensor Integration Package and Test

### 3.1. Sensor Package and Experiment Test

**Figure 9:**
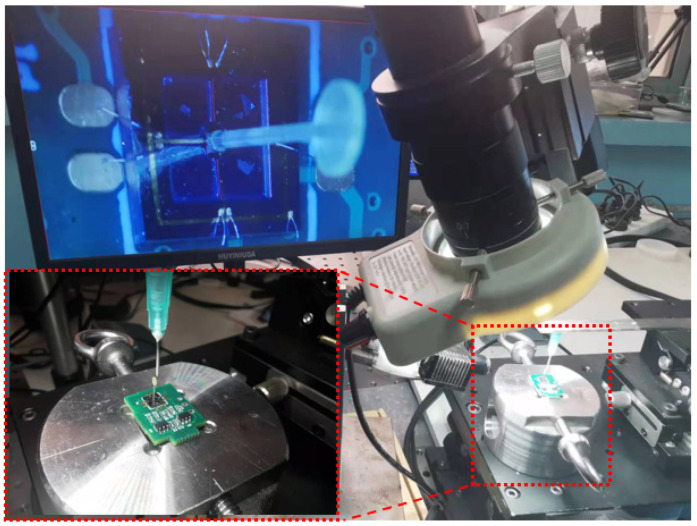
Secondary integration of sensor microstructure.

The bionic cilium and sensor microstructures were secondarily integrated with a UV-cured adhesive. Under the microscopic platform, an ultraviolet-curing adhesive was utilized to combine the cilium with the cantilever beam structure, thereby achieving the sensor microstructure fabrication, as depicted in Figure 9.

Meanwhile, since the sensor’s sound pickup mechanism bases itself on the bionic otic hair cells of humans, a non-contact monitoring design was adopted. It was encapsulated with the common stethoscope membrane body at present, with serious sound loss and low sensitivity. Cardiac sound signals were acoustically quite weak, which may be lethal to the heart sound auscultation process.

**Figure 10:**
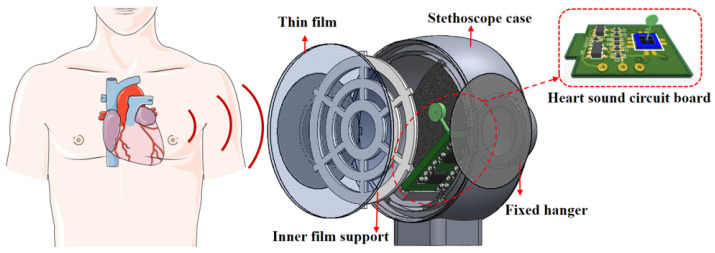
Schematic diagram of acoustic auscultation with bionic heart sound sensor.

The schematic diagram of acoustic auscultation by a bionic sensor is shown in Figure 10. When inputting sound on the boundary between two varying media, the reflection coefficient increases, whereas the transmitted acoustic energy decreases with the heightening impedance difference. Upon contact of the stethoscope with skin, the reflection between them becomes strong, resulting in higher attenuation and less transmitted acoustic energy. Thus, for the reception of the maximum sound signal, the heart sounds were transmitted to the MEMS-based bionic sensor after crossing the human tissues, showing progressive attenuation. In addition, medical silicone oil (20cst) was selected as the encapsulation material by meeting the characteristic acoustic impedance requirements of human soft tissue. Sound velocity and acoustic characteristic impedance of different media can be seen in Table 3.

(6) $$T=\frac{4Z_{1}Z_{3}}{{\left(Z_{3}+Z_{1}\right)}^{2}cos^{2}\left(k_{2}L\right)+{\left(Z_{2}+\frac{Z_{1}Z_{3}}{Z_{2}}\right)}^{2}sin^{2}\left(k_{2}L\right)}$$

**Figure 11:**
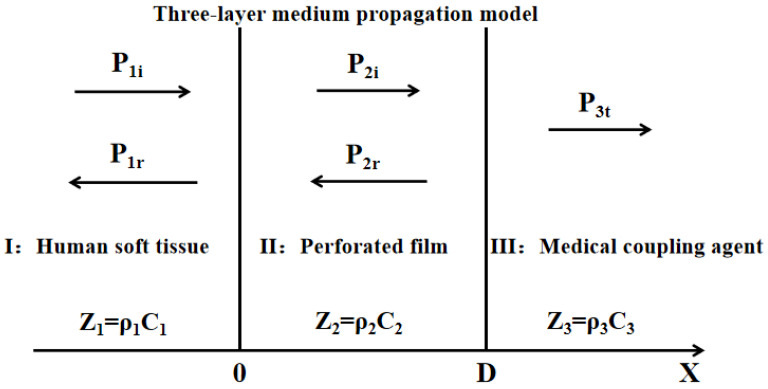
Three-layer medium propagation model.

The three-layer medium propagation model is shown in Figure 11, where Pi and Pr are the acoustic pressure of incident wave and reflected wave, respectively, *Z*_1_, *Z*_2_, and *Z*_3_ refer to the acoustic characteristic impedance of the three-layer medium of human soft tissue, the e-PTFE (expanded polytetrafluoroethylene) porous membrane, and the coupler agent, respectively. *k*_2_ means the number of sound waves transmitting via the e-PTFE medium, and *L* represents the e-PTFE thickness. The coefficient for acoustic wave transmission can approximate *L* only when the acoustic eigen impedance in the three-layer medium is approached, or when the thickness in the intermediate e-PTFE medium is thin enough, according to Formula (6) [16].

**Figure 12:**
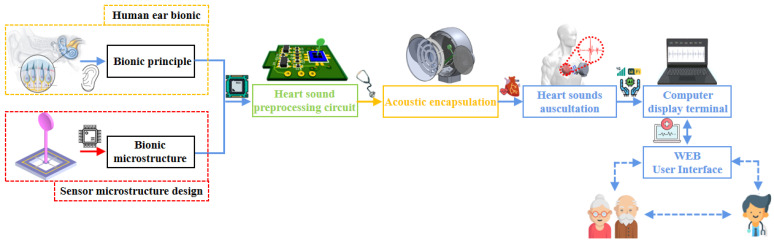
System’s overall design of the block diagram.

**Figure 13:**
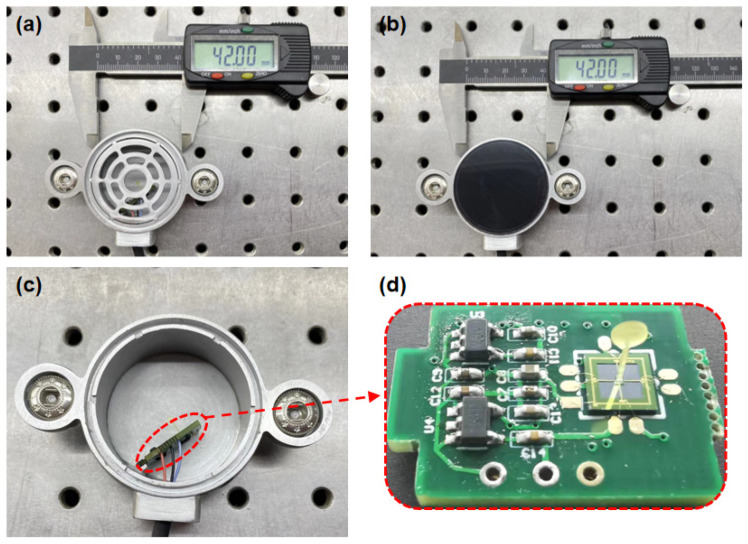
Overall packaging of MEMS heart sound sensor. (**a**) The package structure is supported within the sensor. (**b**) Waterproof and sound-permeable membrane encapsulation. (**c**) Internal structure of bionic MEMS heart sound sensor. (**d**) Heart sound signal preprocessing circuit.

At the same time, the stethoscope probe shell is machined with aluminum alloy, which has high precision, and a light-weight and polished surface, which reduces the interference and influence of external environmental noise on the sensor to a certain extent. The overall design of the block diagram of the system is shown in Figure 12, and the sensor package picture is shown in Figure 13.

**Figure 14:**
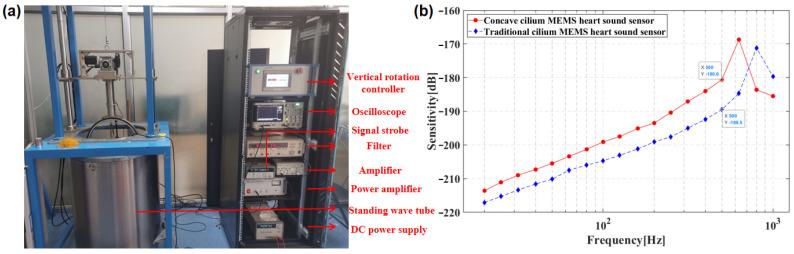
(**a**) Test of the standing wave tube calibration system. (**b**) Comparison diagram of sensor sensitivity test curve.

The standing wave barrel calibration system was adopted to test the integrated concave ciliated MEMS heart sound sensor, as shown in Figure 14a,b. The standing wave barrel is a standing wave sound field. According to the sound pressure distribution theory of the standing wave sound field, the sound pressure sensitivity level of the biomimetic MEMS heart sound sensor is given by Equation (7), and the calibration frequency is performed by a 1/3 octave band [13,14].

(7) $$M_{p}=M_{0}+20\log\left(\frac{U_{pgr}sin\left(kd_{0}\right)}{U_{0}cos\left(kd\right)}\right)$$

In Equation (7), $M_{p}$ is the sound pressure sensitivity level of the MEMS heart sound sensor; $M_{0}$ is the sound pressure sensitivity level of the standard sound sensor; $U_{pgr}$ is the open-circuit voltage of the MEMS heart sound sensor; $U_{0}$ is the open-circuit voltage of the standard sound sensor; $d_{0}$ is the water penetration depth of the standard sound sensor; d is the water penetration depth of the bionic MEMS heart sound sensor; *k* is in the wave number; k=2πf/c; *f* is the frequency; and *c* is the speed of sound in the medium. The sensitivity test results of the MEMS test are shown in Figure 13b. The experimental results show that the working bandwidth of the MEMS heart sound sensor with the integrated concave cilium is 20~600 Hz and the sensitivity is −180.6 dB (@500 Hz). The sensitivity of the traditional bionic ciliated MEMS heart sound sensor is −189.5 dB (@500 Hz). The sensitivity of the integrated concave ciliated MEMS heart sound sensor is 8.9 dB (@500 Hz) higher than that of the traditional bionic cilium MEMS heart sound sensor. Therefore, in order to meet the needs of the low-frequency and high-sensitivity heart sound signal detection, this study proposes that the integrated concave cilium MEMS heart sound sensor has more obvious advantages.

### 3.2. Comparison and Analysis of Sensor Performance

**Figure 15:**
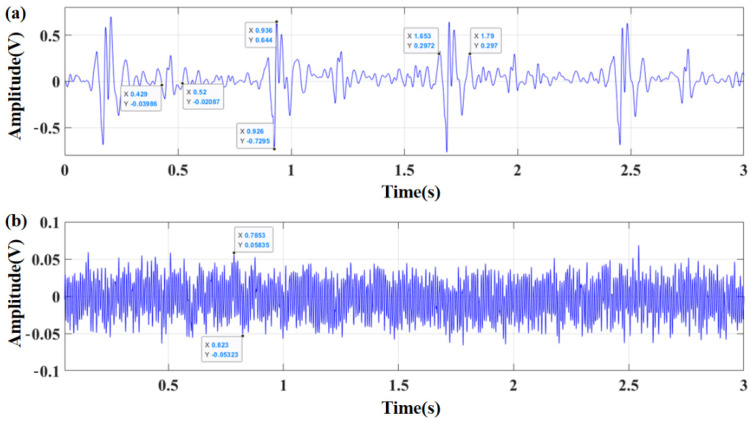
(**a**) The heart sound test results for 3M Littmann 3200 stethoscope; (**b**) the static noise for 3M Littmann 3200 electronic stethoscope.

**Figure 16:**
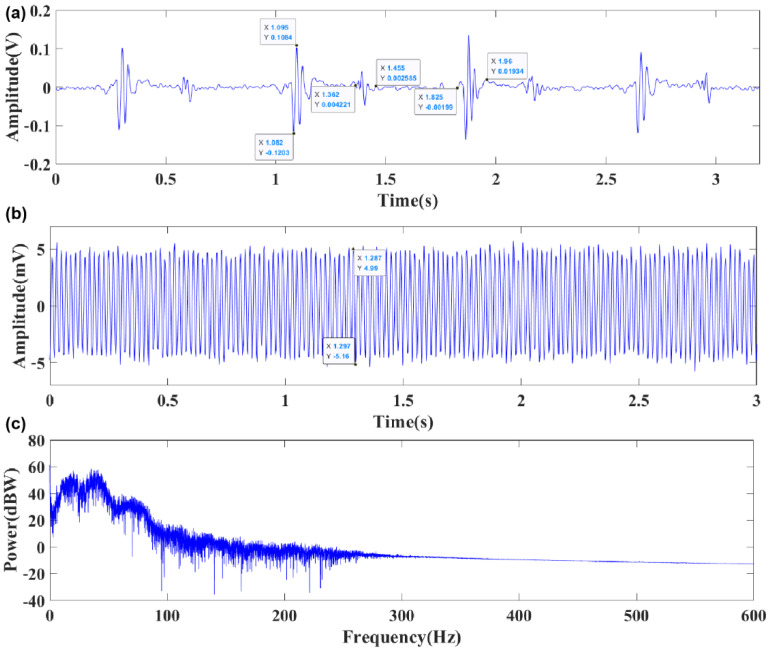
(**a**) The heart sound test results of bionic MEMS heart sound sensor; (**b**) the static noise of bionic MEMS heart sound sensor. (**c**) Frequency spectrum of heart sound signal.

The 3M Littmann electronic stethoscope has wide circulation and high reliability. The test object was a healthy adult male. During the experiment, the 3M Littmann 3200 electronic stethoscope and bionic MEMS heart sound sensor proposed in this paper were chosen for comparison and verification. The heart sound test results and static noise for the 3M Littmann 3200 electronic stethoscope are illustrated by Figure 15a,b. The heart sound test results and static noise for bionic MEMS heart sound sensor is shown in Figure 16a,b. As demonstrated by the experimental results, the signal-to-noise ratios (SNRs) are up to 27.05 dB and 21.80 dB for the MEMS cardiac sound sensor and the 3M electronic stethoscope, respectively. The signal-to-noise ratio of the traditional bionic ciliated MEMS heart sound sensor is 25.62 dB. The signal-to-noise ratio of the MEMS heart sound sensor designed in this paper is 5.25 dB and 1.43 dB higher than that of the 3M electronic stethoscope and the traditional MEMS heart sound sensor, respectively, which proves the feasibility of the MEMS heart sound sensor in this paper to collect heart sound signals. The experimental results also confirm that microstructure design presented in the paper is rational. The signal-to-noise ratio can be calculated using Equation (8), and heart sound signal test data are shown in Table 4.

(8) $$SNR=20\log\left(\frac{V_{S}}{V_{n}}\right)$$

where $V_{S}$ refers to the heart sound output peak, and $V_{n}$ indicates the background noise of the stethoscope.

**Figure 17:**
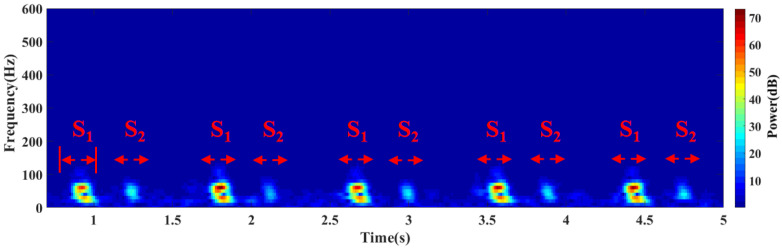
Heart sound signal spectrogram.

With multicomponent and non-stationary traits, the cardiac sound signals are the result of complicated mechanical acoustic phenomena, which contain predictive information and, thus, have a diagnostic value in the clinical setting. Time-frequency analysis was performed on the collected heart sound signals and Figure 16c shows the analysis results of the relevant features [17,18,19,20]. As demonstrated by them, the cardiac sound signal chiefly comprises the first and second cardiac sounds, where the duration of a single cardiac cycle is about 0.8 s. As an acoustic signal, the heart sound signal reflects the most important perceptual characteristics in the spectrum and reveals the dynamic change process of it. The value of the time-frequency analysis on the cardiac sound signal is not only evident for basic research, but also for cardiac system diagnosis in the clinical setting. The results of the spectrum analysis show that the heart sound signal frequency primarily centralizes in 20~150 Hz and there is only weak heart murmur distribution in other frequency bands. The heart sound signal spectrogram is shown in Figure 17. Through the analysis of the spectrogram, it can be seen that the energy of the heart sound signal is mainly concentrated in *S*_1_ and *S*_2_, and it has a periodic dynamic change law [21,22,23].

When designing a heart sound stethoscope, in order to increase the sensitivity, it is usually necessary to reduce the bandwidth but ensure that the frequency band is higher than 600 Hz. Finally, an integrated concave cilium MEMS heart sound sensor was proposed. Through experimental tests, the sensitivity of the integrated concave cilium MEMS heart sound sensor in the 20~600 Hz frequency band was −180.6 dB@500 Hz and the bandwidth was above 600 Hz, which meets the 20~600 Hz working range of the heart frequency distribution.

## 4. Discussion and Conclusions

In view of early digital diagnosis and treatment of cardiovascular disease, an integrated concave cilium MEMS heart sound sensor was proposed based on the bionic principles and piezoresistive principles in this paper; the low-frequency and high-sensitivity characteristics of the MEMS heart sound sensor were realized. Firstly, the pickup principle of the sensory organ structure of the bionic human ear sound was integrated with the propagation characteristics of the human heart sound. This allows the sensor to collect the heart tone signal using a method that has the characteristics of being low cost, and having high sensitivity and portable non-invasive detection. Secondly, the sound-sensitive microstructure was prepared using MEMS technology, and through the design and optimization of the high-efficiency sound transmission acoustic coupling packaging, the sensor had high sensitivity characteristics (−180.6 dB@500 Hz), favorable low-frequency characteristics, and a wide working frequency range (10~630 Hz). Finally, experimental tests and results showed that the signal-to-noise ratio (SNR) of the MEMS heart sound sensor with concave cilium was 27.05 dB, being 5.25 dB and 1.43 dB higher than the 3M Littmann electronic stethoscope and traditional MEMS heart sound sensor, respectively. At the same time, the collected heart sound signals were analyzed to verify the concave ciliated MEMS heart sound sensor had a good heart sound detection ability. Therefore, the sensor structure design proposed in this paper is feasible. In conclusion, the structural design proposed in this paper is of great significance for the early detection and treatment of cardiovascular diseases. At the same time, it also provides a reference for future research.

## Figures and Tables

**Table 1:** Dimensions and parameters.

Parameters	Dimensions	Parameters	Dimensions
Length of cantilever beams	L (1 mm)	Thickness of cantilever beams	t (0.03 mm)
Width of beams	b (0.12 mm)	Mass block length	a (0.3 mm)

**Table 2:** Material attributes.

Material	Density (Kg/m^3^)	Poisson’s Ratio	Young’s Modulus (Pa)
Acrylic resin	1.2 × 10^3^	0.41	4.2 × 10^9^
Beam structure (Si)	2.33 × 10^3^	0.27	1.6 × 10^11^

**Table 3:** Sound velocity and acoustic characteristic impedance of different media.

Medium	Density [ρ (Kg/m^3^)]	Acoustic Characteristic Impedance [z (Pa·s/m)]
Air (20 °C)	1.21	415
Water (20 °C)	998	1.48 × 10^6^
Blood	1055	1.656 × 10^6^
Soft tissue	1016	1.524 × 10^6^
Muscle	1074	1.684 × 10^6^
Medical coupling agent	1016	1.5~1.7 × 10^6^

**Table 4:** Heart sound signal test data.

Parameter	*S*_1_ (ms)	*S*_2_ (ms)	VP-P (mV)	Background Noise (mV)	SNR (dB)
3M electronic stethoscope	137	91	1373.5	111.58	21.80
Traditional bionic ciliated MEMS heart sound sensor	134	105	210	11	25.62
Integrated concave ciliated MEMS heart sound sensor	135	93	228.7	10.15	27.05
